# The Predictive Value of Tenascin‐C, Neopterin and Pro‐Inflammatory Cytokines (IFN‐γ, TNF‐α, IL‐6) in Alcohol Use Disorder Treatment Response

**DOI:** 10.1111/adb.70181

**Published:** 2026-07-23

**Authors:** Guleser Akpinar, Gulsen Sener, Enes Faruk Altunkiliç, Emre Yazgec, Alp Batuhan Ozturk, Nuri Mehmet Basan, Huseyin Aycan Ozsemerci

**Affiliations:** ^1^ Department of Emergency Medicine Basaksehir Cam and Sakura City Hospital Istanbul Turkey; ^2^ Department of Medical Biochemistry Basaksehir Cam and Sakura City Hospital Istanbul Turkey; ^3^ Department of Child and Adolescent Mental Health and Disorders Bakirkoy Prof. Dr. Mazhar Osman Mental Health and Neurological Diseases Training and Research Hospital Istanbul Turkey; ^4^ Department of Emergency Medicine, Faculty of Medicine Biruni University Istanbul Turkey; ^5^ Department of Neurology Istanbul Basaksehir Cam and Sakura City Hospital Istanbul Turkey

**Keywords:** alcohol use disorder, neopterin, psychiatric symptoms, tenascin‐C

## Abstract

The underlying neurobiological mechanisms of chronic alcohol consumption, particularly neuroinflammation and immune system dysregulation, have been extensively studied in recent years. In this process, the identification of objective biomarkers that can predict treatment response and enable personalized medicine approaches is of critical importance. This study aimed to evaluate the potential predictive value of tenascin‐C (TNC), an extracellular matrix protein, neopterin, a marker of cellular immune activation and pro‐inflammatory cytokines interferon‐gamma (IFN‐γ), tumour necrosis factor‐alpha (TNF‐α) and interleukin‐6 (IL‐6), during the treatment process for alcohol use disorder (AUD). This prospective observational cohort study included 30 patients admitted to an AUD treatment centre and 31 healthy controls. Serum TNC, IL‐6, TNF‐α and IFN‐γ levels were measured using the ELISA method. Clinical outcomes were assessed using the SCL‐90‐R and API‐K scales. Serum samples were collected from patients at admission and on the 7th day of detoxification treatment. Baseline serum TNC levels in the alcohol group (131.78 ± 50.92 ng/mL) were significantly higher than in the healthy control group (63.92 ± 23.85 ng/mL). In the alcohol group, TNC (*p* = 0.007) and AST (*p* = 0.025) levels decreased significantly, while vitamin B12 (*p* = 0.002) and platelet count (*p* = 0.012) increased. No significant difference was observed in neopterin and cytokine levels (TNF‐α, IL‐6 and IFN‐γ) between baseline and follow‐up measurements. Clinical assessments showed significant improvements in depression (*p* = 0.041), anxiety (*p* = 0.014) and total symptom scores (*p* = 0.026) on the SCL‐90‐R scale. ROC analysis showed that a reduction of 28.22% or more in TNC predicted clinical improvement with an overall accuracy rate of 73.33%.

## Graphical abstract

Thirty patients with AUD undergoing detoxification and 31 healthy controls were evaluated. From baseline to day 7, TNC decreased significantly, accompanied by improvements in depression, anxiety and total symptom scores. Neopterin and cytokine levels remained unchanged. A TNC reduction of ≥28.22% predicted clinical improvement with 73.33% accuracy, supporting TNC as a candidate biomarker for monitoring treatment response in AUD

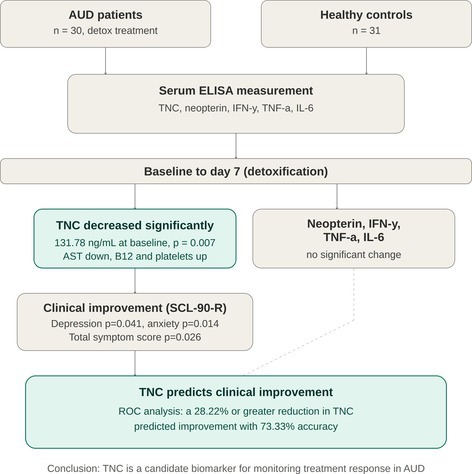

## 1 Introduction

Alcohol use disorder (AUD) is a major public health problem worldwide due to its biological, psychological and social effects. This condition also has a negative impact on mental health, interpersonal relationships and overall life satisfaction [1, 2].

Alcohol exerts direct neurotoxic and neuroinflammatory effects on the central nervous system and affects brain function beyond transient cognitive impairments. These effects may lead to permanent neurocognitive impairments over time. Furthermore, alcohol consumption can trigger the development of psychiatric disorders such as depression, bipolar disorder and psychosis, while also exacerbating the progression of existing mental health conditions [3, 4, 5].

The assessment of alcohol‐induced inflammatory processes through biochemical approaches has emerged as a promising strategy for both diagnostic and treatment monitoring purposes [6, 7].

Given these widespread effects, the diagnosis and treatment of AUD are of vital importance [8, 9].

The pro‐inflammatory mediator neopterin is recognized as a biomarker of cellular immunity. It is released by monocytes/macrophages when activated by interferon‐gamma (IFN‐γ); tumour necrosis factor‐alpha (TNF‐α) and endotoxins can also co‐stimulate neopterin production. Changes in neopterin levels reflect the immune system's response to treatment and the resolution of neuroinflammatory processes. Consequently, neopterin levels possess both diagnostic and prognostic value [10, 11, 12].

Molecules involved in tissue damage and repair processes are of critical importance in understanding neuroimmune interactions. In this context, it is known that the expression of Tenascin‐C (TNC), a dynamic glycoprotein of the extracellular matrix (ECM), is increased in conditions such as inflammation, wound healing and neuronal plasticity [13, 14]. Recent studies have shown that TNC modulates neuroinflammatory processes and that differences in its serum levels are observed in various neurological and psychiatric disorders [15, 16].

The aim of this study was to evaluate changes in pre‐ and post‐treatment serum levels of TNC, neopterin and pro‐inflammatory cytokines (TNF‐α, interleukin‐6 (IL‐6) and IFN‐γ) in individuals diagnosed with AUD; furthermore, to investigate the relationship between these biomarkers and psychiatric symptoms and treatment response.

## 2 Methods

This study was conducted using serum samples collected and appropriately stored as part of a research project approved by the Ethics Committee of Bakirkoy Mazhar Osman Mental Health and Neurological Diseases Training and Research Hospital (Approval No: 221909003). Additional ethical approval for the biomarker analyses performed on the stored serum samples was obtained from the Biruni University Non‐Interventional Clinical Research Ethics Committee (Approval No: 2024‐BIAEK/20‐15). Written informed consent, including permission for the use of biological samples in current and future related research, was obtained from all participants. The study was conducted in accordance with the ethical principles of the Declaration of Helsinki.

Inpatients diagnosed with AUD according to the criteria in the ‘Diagnostic and Statistical Manual of Mental Disorders, Fifth Edition (DSM‐5)’ were included in the study. The inclusion criterion for the patient group was individuals aged 18 years and over who had been diagnosed with isolated AUD according to the DSM‐5 diagnostic criteria [17]. Exclusion criteria included the use of steroids, probiotics, antibacterial agents or non‐steroidal anti‐inflammatory drugs within the 2 months prior to the study; substance misuse; a body mass index (BMI), metabolic disorders such as diabetes, chronic inflammatory diseases, cancer, HIV, neurodegenerative diseases, pregnancy or neurological disorders. A total of 61 participants were included in the study. The study groups consisted of an AUD group comprising 30 patients and a control (C) group comprising 31 healthy adults. The C group consisted of healthy volunteers aged 18 years and over with no history of disease and no regular medication use in the previous 6 months. The healthy control group underwent a single blood sample collection on an empty stomach in the morning at the time of study enrollment, and no long‐term follow‐up was conducted. Blood samples were collected from participants on an empty stomach in the morning (after fasting for at least 8 h) prior to the start of treatment and 1 week after treatment. Biochemical analyses included blood ethanol levels, alanine aminotransferase (ALT) and aspartate aminotransferase (AST) activities, hepatitis markers, leukocyte, erythrocyte and platelet counts, haemoglobin, haematocrit, mean cell volume (MCV), vitamin B12 and folate levels, which were evaluated by the hospital biochemistry laboratory.

Serum samples were separated into Eppendorf tubes after centrifugation at 3000 rpm for 10 min and stored at −80°C until analysis. Serum TNC levels were measured using a commercial ELISA kit (Sunred, Catalogue number: E‐EL‐H6198; Sensitivity: 26.15 pg/mL; Detection Range: 78.13–5000 pg./mL; intra‐assay CV: < 10%; inter‐assay CV: < 10%).

Measurements of inflammatory markers IL‐6, IFN‐γ, neopterin and tumour necrosis factor‐alpha (TNF‐α) were performed using a commercial enzyme‐linked immunosorbent assay (ELISA) method according to the manufacturer's kit procedures. Human Interleukin 6 (IL‐6) BT Lab ELISA Kit (E0090Hu), Human Interferon Gamma (IFN‐γ) BT Lab ELISA Kit (E0105Hu), Neopterin IBL ELISA Kit (RE59321) and Human Tumour Necrosis Factor a (TNF‐α) BT Lab ELISA Kit (E0082Hu) [18].

Psychiatric assessment was performed using the Addiction Profile Index Clinical Form (API‐K) and Symptom Checklist‐90‐Revised (SCL‐90‐R) scales. The API‐K scale measures six different clinical domains and consists of subscales for depression, anxiety, lack of anger control, lack of safe behaviour, thrill‐seeking behaviour and impulsivity. The SCL‐90‐R scale consists of 90 questions measuring nine core symptom dimensions and assesses somatization, obsessive‐compulsive, interpersonal sensitivity, depression, anxiety, hostility, phobic anxiety, paranoid ideation and psychoticism [19, 20].

Inpatient Treatment and Withdrawal Management: All participants with AUD received standard inpatient treatment at the AMATEM unit during the detoxification and withdrawal management period. As part of standard inpatient care, patients received acamprosate for relapse prevention together with oral vitamin B12 (1000 μg/day) and riboflavin (25 mg/day) supplementation to correct alcohol‐related nutritional deficiencies. Information regarding medication use, duration of hospitalization and relevant clinical characteristics was obtained from medical records and incorporated into the descriptive analyses.

### 2.1 Statistical Analysis

All statistical analyses were performed using IBM SPSS Statistics (version 26.0) and JAMOVI. The distribution of continuous variables was assessed using the Shapiro–Wilk test. Continuous variables with a normal distribution were reported as mean ± standard deviation (M ± SD), whereas non‐normally distributed variables were reported as median (Q1–Q3). Categorical variables were summarized as *n* (%).

For comparisons between two independent groups (control vs. alcohol group), the independent‐samples *t* test was used when the assumptions of normality and homogeneity of variances were met. When the homogeneity of variance assumption was violated, Welch's *t* test was reported. For continuous variables not meeting the normality assumption, the Mann–Whitney *U* test was applied and the test statistic was reported as a *Z* value. Categorical variables were compared using the chi‐square test or Fisher's exact test, as appropriate based on expected cell counts.

Within the alcohol group, pre–post (repeated‐measures) comparisons were conducted using the paired‐samples *t* test for normally distributed variables and the Wilcoxon signed‐rank test for non‐normally distributed variables; the corresponding test statistics were reported as *t* or *Z* values, respectively.

Because a significant pre–post difference was observed only for the TNC parameter, the discriminative performance of the percentage change in TNC for identifying a decrease in the ‘SCL general symptom score’ was evaluated using receiver operating characteristic (ROC) analysis. Discrimination was reported as the area under the curve (AUC) with 95% confidence intervals. The optimal cut‐off value was determined based on the Youden index, and sensitivity, specificity and accuracy at the selected cut‐off were calculated.

All tests were two‐sided, and statistical significance was set at *p* < 0.05.

## 3 Results

This prospective observational cohort study included 30 patients admitted to an AUD treatment centre and 31 healthy controls. When the groups were compared sociodemographically, the male gender ratio was significantly higher in the alcohol group (*p* = 0.001). The age of the alcohol group was found to be higher than that of the control group (*p* < 0.001). No significant difference was observed between the groups in terms of HBsAg and Anti‐HCV positivity (*p* > 0.05) (Table 1).

**TABLE 1:** Comparison of sociodemographic data of control and patient groups.

Variables	Control (*n* = 31)	Alcohol (*n* = 30)	Test	*p*
	M ± SD/*n* (%)	M ± SD/*n* (%)		
Gender				
*Male*	18 (58.1)	29 (96.7)	10.76^a^	**0.001**
*Female*	13 (41.9)	1 (3.3)		
Age	41.55 ± 8.00	50.63 ± 8.41	−4.32^b^	< 0.001
Height (cm)	170.48 ± 8.59	174.00 ± 9.81	−1.49^b^	0.141
Weight (kg)	79.61 ± 15.38	76.07 ± 14.86	0.92^b^	0.364
BMI	27.29 ± 4.32	24.99 ± 3.52	2.28^b^	0.026
Educational status				
*Primary*	0 (0.0)	11 (36.7)	28.2^c^	< 0.001
*Secondary school*	2 (6.5)	4 (13.3)		
*High School*	5 (16.1)	11 (36.7)		
*University*	24 (77.4)	4 (13.3)		
Duration of hospitalization (days)	—	12.0 (8.0–21.0)		
Duration of substance use (years)	—	25 (17–34.5)		
Amount of substance use, day	—	850 (500–1000)		
Presence of chronic disease	1 (3.2)	6 (20.0)	4.22^c^	0.053
Medication use	0 (0)	10 (33.3)	12.4^c^	< 0.001
Presence of additional psychiatric illness	0	7 (23.3)	8.17^c^	0.005
Use of psychiatric drugs	0	13 (43.3)	17.1^c^	< 0.001
HBsAg positivity	1 (3.2%)	5 (16.7%)	3.11^c^	0.104
Anti‐HCV positivity	0 (0%)	1 (3.3%)	1.05^c^	0.492

Abbreviations: M: Mean. SD: Standard deviation.

^a^ Continuity correction.

^b^ Students *t*‐test.

^c^ Fishers exact test.

When the blood parameters of the control group and the alcohol group were compared at admission, the alcohol‐admission group had lower RBC levels (*p* = 0.004) and higher TNC, MCV and B12 levels (*p* < 0.001, *p* < 0.001 and *p* = 0.042, respectively) (Table 2).

**TABLE 2:** Comparison of blood parameters at admission and discharge in the clinical group.

Variables	Alcohol‐admission M ± SD/Med. (Q1–Q3)	Alcohol‐discharge M ± SD/Med. (Q1–Q3)	Test	*p*
ALT	26.5 (14.3–40.8)	22.0 (17.3–30.8)	−1.07^b^	0.284
AST	24.5 (20.3–54.3)	23.0 (19.0–26.0)	−2.24^b^	0.025
WBC	8.14 ± 2.82	8.76 ± 2.68	−1.51^a^	0.142
RBC	4.49 ± 0.46	4.52 ± 0.47	−0.58^a^	0.567
PLT	235.60 ± 79.21	273.07 ± 71.99	−2.68^a^	0.012
HGB	13.9 (12.4–14.9)	13.9 (12.3–14.6)	−1.44^b^	0.151
HCT	44.8 (40.9–46.0)	43.3 (39.7–45.2)	−2.27^b^	0.023
MCV	95.4 (90.3–99.6)	94.2 (90.4–97.2)	−3.05^b^	0.002
B12	450 (347–630)	664 (397–789)	−3.12^b^	0.002
Folic acid	6.55 (3.97–20.0)	7.90 (4.90–20.0)	−1.77^b^	0.077
Neopterin	0.921 (0.682–1.15)	0.846 (0.679–0.997)	−0.81^b^	0.417
TNF‐alpha	102 (84.6–156)	92.7 (65.3–132)	−1.30^b^	0.194
IL‐6	132.0 (67.4–144.0)	82.6 (71.4–134.0)	−0.98^b^	0.329
Interferon‐gamma	37.8 (23.2–57.4)	40.8 (36.7–66.6)	−1.27^b^	0.206
TNC	131.78 ± 50.92	100.47 ± 44.34	2.93^a^	0.007

Abbreviations: M: Mean, Med.: Median, Q1–Q3: First and third quartiles, SD: Standard deviation.

^a^ *t* test.

^b^ Wilcoxon (*z*).

When comparing the blood parameters of the control group with those of the alcohol‐exit group, TNC, B12, MCV and WBC were found to be higher in the alcohol group (*p* < 0.001, *p* < 0.001, *p* < 0.001 and *p* = 0.003, respectively), while RBC was lower (*p* = 0.010). Folic acid levels were also higher in the alcohol group (*p* = 0.019).

In the alcohol group, statistically significant decreases were observed in MCV, TNC, HCT and AST values in the admission–discharge comparisons (*p* = 0.002, *p* = 0.007, *p* = 0.023 and *p* = 0.025, respectively). B12 and PLT levels were significantly increased at discharge (*p* = 0.002, *p* = 0.012). No significant differences were observed between admission and discharge for ALT, WBC, RBC, HGB, folic acid, neopterin, TNF‐α, IL‐6 and IFN‐γ (all *p* > 0.05).

When comparing API‐K scores between the control group and the alcohol‐admission group, the alcohol group had higher Lack of Safe Behaviour scores (*p* = 0.002) and significantly higher API‐K Depression and API‐K Anxiety scores (both *p* < 0.001). No significant differences were found between the groups in the sub‐dimensions of Inadequate Anger Control, Thrill‐Seeking Behaviour and Impulsivity (all *p* > 0.05). In the alcohol group, no significant changes were observed in the majority of variables assessed in the pre–post comparison of API‐K subscales and total scores (all *p* > 0.05). Although a small decrease was observed in the mean score of the effects on life subscale at post‐treatment, this change was not statistically significant (*p* = 0.723).

At baseline assessment, the SCL subscale scores of the alcohol group were generally higher than those of the control group. Initially, the alcohol group showed significant increases in total symptom scores, including somatization (*p* = 0.003), obsessive‐compulsive symptoms (*p* = 0.001), interpersonal sensitivity, depression, anxiety, hostility, phobic anxiety, paranoid ideation, psychoticism, supplementary scales and total symptom scores (all remaining *p* < 0.001).

At discharge, the alcohol group continued to exhibit significantly higher SCL‐90‐R scores than the control group, including obsessive‐compulsive symptoms (*p* = 0.002) and paranoid ideation (*p* = 0.004), while all remaining domains were significant at *p* < 0.001. No significant difference was observed between the groups in the somatization subscale (*p* = 0.080).

Treatment was associated with significant improvements in several SCL‐90‐R domains. Significant reductions were observed in depression (*p* = 0.041), anxiety (*p* = 0.014), additional scale scores (*p* = 0.035) and global symptom severity (*p* = 0.026). No significant changes were observed in somatization, obsessive‐compulsive symptoms, interpersonal sensitivity, hostility, phobic anxiety, paranoid ideation or psychoticism (all *p* > 0.05). Psychoticism showed a borderline improvement (*p* = 0.051) (Table 3).

**TABLE 3:** Comparison of SCL scale scores at admission and discharge in the clinical group.

Variables	Alcohol‐admission M ± SD/Med. (Q1–Q3)	Alcohol‐discharge M ± SD/Med. (Q1–Q3)	Test	*p*
SCL‐Somatic	1.42 (0.79–1.83)	1.17 (0.52–1.75)	−1.73^b^	0.083
SCL‐OCD	1.56 ± 0.82	1.45 ± 0.73	1.28^a^	0.212
SCL‐KAD	1.35 ± 0.96	1.20 ± 0.78	1.28^a^	0.211
SCL‐Dep.	1.58 ± 0.89	1.32 ± 0.72	2.13^a^	0.041
SCL‐Anxiety	1.30 (0.80–1.60)	1.10 (0.73–1.37)	−2.46^b^	0.014
SCL‐ÖD	1.14 ± 0.91	1.00 ± 0.72	1.62^a^	0.116
SCL‐FA	0.57 (0.18–1.10)	0.57 (0.14–0.71)	−1.88^b^	0.060
SCL‐Paranoid	1.33 (0.58–1.67)	1.17 (0.50–1.67)	−1.62^b^	0.106
SCL‐Psych.	0.60 (0.50–1.17)	0.55 (0.30–0.88)	−1.96^b^	0.051
SCL‐ES	1.93 (1.29–2.29)	1.71 (0.90–2.10)	−2.11^b^	0.035
SCL‐GS	1.35 (0.90–1.60)	1.15 (0.70–1.52)	−2.23^b^	0.026

Abbreviations: Anx: Anxiety, Dep: Depression, EK: Additional scales, FA: Phobic anxiety, GS: General symptoms, H: Hostility, HAD: Interpersonal sensitivity, M: Mean, Med: Median, OCD: Obsessive‐compulsive symptoms, PD: Paranoid ideas, PSY: Psychoticism, Q1–Q3: 1st and 3rd quartiles (interquartile range), SCL: Symptom Checklist, SD: Standard deviation, Som: Somatization.

^a^ *t* test.

^b^ Wilcoxon test (*z*).

**FIGURE 1:**
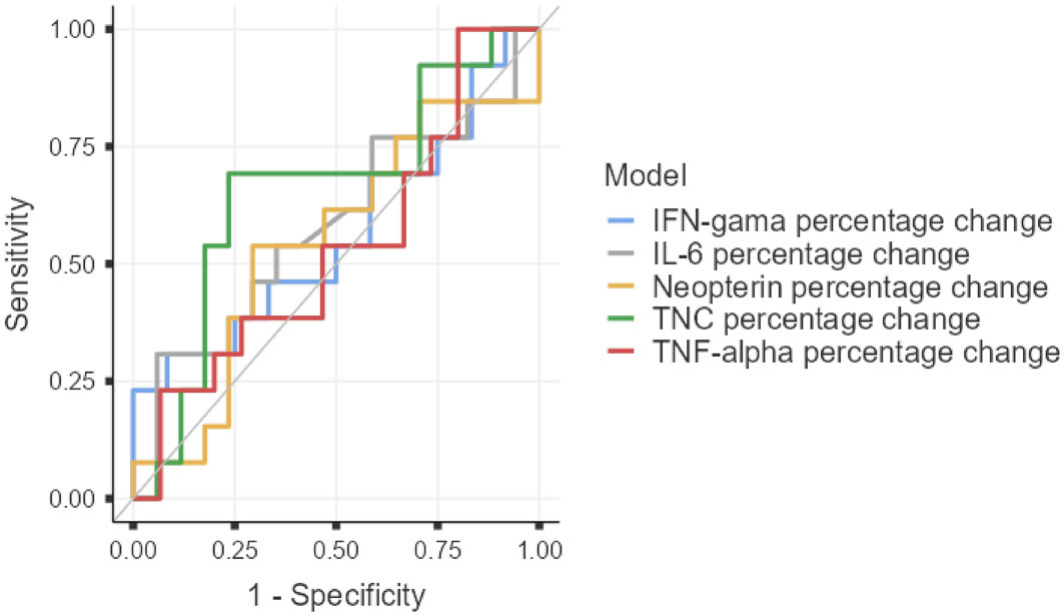
Performance of SCL general symptom score in distinguishing cases with decreasing entry–exit percentage change parameters (ROC analysis).

The performance of the entry–exit percentage change parameters in distinguishing cases with decreasing SCL total symptom scores was evaluated using ROC analysis. The AUC values were 0.55 (95% CI: 0.33–0.77; *p* = 0.673) for neopterin % change, 0.53 (95% CI: 0.31–0.76; *p* = 0.772) for TNF‐α % change, 0.58 (95% CI: 0.36–0.80; *p* = 0.480) for IL‐6 percentage change, IFN‐γ % change was 0.56 (95% CI: 0.33–0.80; *p* = 0.593) and TNC % change was 0.66 (95% CI: 0.45–0.87; *p* = 0.148). Overall, the AUC values of the biomarkers examined ranged from 0.53 to 0.66, showing no statistically significant discriminatory power (all *p* > 0.05) (Figure 1).

## 4 Discussion

This study examined the effects of AUD treatment on biochemical and psychiatric parameters using immunological markers. Our findings indicate that AUD is not merely a behavioural disorder, but is also characterized by neuroinflammatory changes affecting the central nervous system.

The main finding of our study is that TNC levels are elevated at baseline in individuals with AUD and decrease significantly following short‐term detoxification treatment. This finding suggests that TNC may be a biomarker of alcohol‐induced cellular damage and the inflammatory response.

Recent studies have reported that ECM proteins such as TNC increase rapidly in neuroinflammatory environments and decrease once tissue stability is restored [13, 16]. This supports that TNC may serve as a marker for monitoring treatment response.

Neopterin is produced by macrophages activated by IFN‐γ and is considered a marker of cellular immune activation [21]. Although the literature indicates that neopterin and IFN‐γ reflect the restoration of cellular immunity, no significant changes in these parameters were observed in our study. This may reflect that a 7‐day detoxification period may be insufficient to correct the immune dysregulation caused by alcohol. The short half‐lives of cytokines and the inhibitory effect of alcohol on cytokine production limit the sensitivity of these molecules in treatment monitoring compared to TNC [22]. Cytokines such as TNF‐α and IL‐6, which were planned to be measured in the research protocol, are central actors in the inflammatory cascade caused by alcohol. The literature supports the view that chronic alcohol use increases pro‐inflammatory cytokines, contributing to both organ damage and mood disorders such as depression and anxiety [23]. In our study, the marked reduction in symptoms of depression and anxiety observed following treatment may be related to the resolution of underlying neuroinflammatory processes. The elimination of alcohol's toxic effects through treatment may have led to a reduction in inflammatory cytokine production and, consequently, exerted a positive effect on mood.

Psychometric assessments confirm that the group of alcohol‐dependent patients exhibited severe psychiatric symptoms at the start of treatment. The concurrent improvement in psychiatric symptoms, particularly depression and anxiety, together with the reduction in TNC levels following treatment supports the notion that TNC may reflect biological changes associated with recovery. These findings indicate that TNC may serve as an early biological marker of treatment‐related improvement in individuals with AUD. This observation shows that biological and psychological mechanisms may be interconnected. A reduction in neuroinflammation may facilitate the re‐regulation of neurotransmitter systems and, consequently, an improvement in mood [24].

Tenascin‐C may contribute to a more objective assessment of the recovery process in the treatment of AUD. While neopterin and cytokines remain stable during the early stages of detoxification treatment, the marked changes in TNC make it stand out in clinical monitoring. Future studies may clarify whether TNC can be incorporated into treatment monitoring protocols.

The simultaneous observation of a reduction in TNC levels alongside an improvement in SCL‐90‐R total symptom scores suggests that TNC may not only be a marker of tissue damage but also a biological marker for monitoring the treatment process. The reorganization of neurotransmitter systems alongside a reduction in neuroinflammation may be considered a possible mechanism explaining the improvement in mood and anxiety symptoms.

In addition to inflammatory biomarkers, changes were also observed in several biochemical parameters during the detoxification period. Following treatment, serum AST levels decreased significantly. AST is recognized as a sensitive marker of alcohol‐related hepatic and extrahepatic tissue damage and has been shown to decrease rapidly following alcohol withdrawal [25]. The reduction in AST observed in our study appears to reflect early improvement in alcohol‐induced tissue damage accompanying abstinence.

A significant increase in serum vitamin B12 concentrations was observed during hospitalization. Chronic alcohol consumption is frequently associated with impaired nutritional status, gastrointestinal dysfunction and altered hepatic vitamin storage. The restoration of a regular diet, vitamin supplementation during hospitalization and improved intestinal absorption may explain the rapid increase in serum vitamin B12 levels observed following detoxification [26, 27].

Similarly, thrombocytopenia is frequently observed in patients with AUD because of bone marrow suppression, splenic sequestration and reduced platelet production. Recovery of platelet counts usually begins within the first week of abstinence, consistent with the increase observed in our study [28]. These findings demonstrate that early detoxification is accompanied not only by improvements in neuroinflammatory markers but also by recovery of hepatic, haematological and nutritional parameters.

This study has certain limitations. The relatively small sample size, the follow‐up period limited to just 7 days and a sample consisting largely of male patients limit the generalizability of the findings. Furthermore, the single‐centre nature of the study and the failure to assess long‐term clinical outcomes and relapse rates are significant limitations. Future studies should examine the prognostic value of TNC in greater detail using larger, longer‐term and multi‐centre designs.

## 5 Conclusion

This study examined the effects of AUD treatment on biochemical and psychiatric parameters, particularly in the context of immune markers such as TNC and neopterin. Tenascin‐C may represent a promising biomarker reflecting the biological changes that occur during the treatment of AUD. Although no significant changes were observed in neopterin and cytokines during the short‐term detoxification period, the significant decrease in TNC suggests that this molecule may play a potential role in monitoring early treatment response.

## Author Contributions

G.A., A.B.O. and G.S. designed and planned the study, conducted the literature review, and managed the data, including quality control. E.Y. and E.F.A. supervised the conduct of the study and the data collection process. N.M.B. and H.A.O. analysed the data. G.A. and A.B.O. are responsible for the final version of the manuscript.

## Funding

The authors have nothing to report.

## Ethics Statement

Ethical Approval: Approved by the Biruni University Non‐Interventional Clinical Research Ethics Committee (Approval No: 2024‐BIAEK/20‐15; 27 April 2026). The study was conducted in accordance with the ethical principles of the Declaration of Helsinki.

## Conflicts of Interest

The authors declare no conflicts of interest.

## Data Availability

The datasets generated and/or analysed during the current study are not publicly available due to ethical and privacy restrictions but are available from the corresponding author upon reasonable request.
